# The effect of using denture adhesives on patient satisfaction with complete dentures; a randomized clinical trial

**DOI:** 10.1186/s12903-023-03757-7

**Published:** 2023-12-19

**Authors:** Nadia S. Ereifej, Yara G. Oweis, Motasum Abu-Awwad

**Affiliations:** 1 Department of Prosthetic Dentistry, Faculty of Dentistry, The University of Jordan, Amman, Jordan; 2 Department of Restorative Dentistry, Faculty of Dentistry, The University of Jordan, Amman, Jordan; 3 Department of Prosthetic Dentistry, Faculty of Dentistry, The University of Jordan, Amman, Jordan

**Keywords:** Denture adhesive, Complete edentulism, Denture retention, Patient satisfaction

## Abstract

**Background:**

Denture adhesives can be useful in improving patients’ satisfaction with complete dentures. However, comparison clinical trials are lacking. The purpose of this randomized clinical trial was to assess the satisfaction of edentulous patients and their oral health impact profile when provided with 3 types of denture adhesives.

**Methods:**

Sixty-four completely edentulous patients seeking complete dentures for their first time were randomly divided into 3 groups. Each group received a set of complete dentures, which were adjusted at review appointments until participants reported no complaints. After 1 month of using the dentures, participants rated their overall satisfaction and their satisfaction regarding comfort, retention, stability, and efficiency of mastication and speech on a 100-mm visual analog scale (VAS). Participants also filled out the oral health impact profile for edentulous patients (OHIP-EDENT) questionnaire. Each group was then given 1 type of denture adhesive to use. Group C received Corega Ultra denture fixative cream (GlaxoSmithKline), Group O received Olivafix (Bonyf), and Group S received Sea. Bond adhesive strips (Sea.Bond). Mann-Whitney U test was used to analyze the differences in VAS scores before and after using the adhesive within each group and Wilcoxon-signed rank test was used to compare OHIP scores and total OHI*P* values before and after using the adhesive within each group (*p* = 0.05). Furthermore, Kruskal Wallis test was used to compare the differences before and after using the adhesives in VAS and OHIP values between the 3 groups.

**Results:**

Significantly higher VAS values were detected in all groups and significantly lower values for many OHIP items in addition to total OHIP values were detected in all groups after using the adhesives (*P* < 0.05), except for the ease of cleaning for Group O and Group S (*P* > 0.05). No significant differences were found in VAS and OHIP values between the 3 groups (*P* > 0.05), except for the ease of cleaning which was significantly different between Group C and Group S (*p* = 0.005).

**Conclusions:**

Using denture adhesives for completely edentulous patients resulted in higher patient satisfaction as indicated by higher VAS scores as well as improved quality of life as indicated by lower OHIP-EDENT scores after using the adhesives. These improvements were not dependent on the type of adhesive, except for ease of cleaning as adhesive strips were easier to clean than paste type adhesives.

**Trial registration:**

This trial was registered at ClinicalTrials.gov (ID: NCT05496283) on 11/08/2022.

## Background

Complete edentulism is one of the major challenges facing the elderly populations. The majority of these patients experience difficulties with different oral functions including chewing, eating, and speaking. These difficulties can profoundly affect their oral health-related quality of life [1, 2]. Conventional complete dentures remain the main treatment option for numerous edentulous patients, especially those constrained by financial or medical limitations [1, 3–6]. Successful treatment with complete dentures depends on the integration of the dentures with the functions of the masticatory system and the psychological acceptance of the patient [3, 7, 8]. This can be affected by many factors such as patients’ age, previous denture wearing experience, expectations, aesthetics, residual ridge form and denture quality [8, 9]. Therefore, patients frequently report various issues, including challenges related to retention and stability of the dentures, difficulties in chewing, and a diminished quality of life and satisfaction [3, 4, 10].

The retention of complete dentures depends on the the interaction of various physical mechanisms, including adaptation to the supporting tissues, the presence of thin film of saliva between the intaglio of the denture and tissues, adequate peripheral edge extension, and atmospheric pressure [11, 12]. However, this retention can suffer due to alterations in both hard and soft tissues, declines in saliva consistency and volume due to age or medication, and changes in neuromuscular control [10, 12, 13]. Among the available options to enhance retention and stability of complete dentures are denture adhesives.

Denture adhesives are commercially available, nontoxic, nonmedical soluble products that have attracted more attention since guidelines for their use were published in 2019 by the Oral Health Foundation [14–16]. Typically, these adhesives consist of a combination of of short- and long-acting synthetic hydrophilic polymers that adhere to the glycoproteins present in the oral mucosa forming a viscous layer that improves the adhesive and cohesive properties between the oral mucosa and the dentures [3, 10, 16]. Denture adhesives can be divided into 2 main types: soluble or insoluble. Insoluble products are made of wax-impregnated cotton cloth with adhesive ingredients, while soluble products include creams, powders, and strips formulated using a mix of fast-solubility and low-solubility polymers or blended partial salts [17, 18].

Numerous studies have consistently demonstrated that denture adhesives help improve the retention and stability of complete dentures, improving the quality of life and satisfaction of patients [2, 4, 11, 13, 15, 16, 18–21]. Their use has been linked to a reduction in the likelihood of tissue trauma and an improved ability to chew and speak effectively. Moreover, these adhesives serve to seal off food particles, potentially augmenting digestion and contributing to the overall health of individuals [6, 12, 16, 18, 21]. They can also serve as a psychological measure for patients who experience difficulty adapting to the treatment, especially soon after denture delivery [3, 7, 10, 12, 22].

Although denture adhesives are widely accepted by patients, dental professionals have been reluctant to endorse them. Patients may use denture adhesives indiscriminately without proper prescription by the dentist, leading to dissatisfaction [4, 12]. Some within the dental community perceive the need for denture adhesives as a reflection of clinical or laboratory oversights [3, 9, 12]. Furthermore, patients have reported that some of these products are messy and may cause gagging [15]. The clinical advantages of using complete denture adhesives are still a topic of debate, mainly due to the limited data available on their effectiveness [10].

The aim of this randomized clinical trial was to compare the reported outcomes and oral health impact profile for edentulous patients before and after using 3 different types of denture adhesives. The null hypotheses were that there would be no significant differences in patient satisfaction, as measured by visual analog scale (VAS) scores or oral health impact profile for edentulous patients (OHIP-EDENT) scores before and after using each type of adhesives, and that these differences would not be significant between the 3 types of adhesives.

## Methods

This study was designed as a randomized, single-blind, clinical trial. The study was conducted in accordance with the World Medical Declaration of Helsinki and conformed to the Consolidated Standards of Reporting Trials (CONSORT) statement for randomized clinical trials [23]. The protocol was reviewed and approved by the deanship of academic research at the university hospital (IRB number: 42–2022) and registered on 11/08/2022 at ClinicalTrials.gov (ID: NCT05496283).

All participants were completely edentulous patients seeking new complete dentures at the prosthodontics specialty clinics at a University Hospital. Only participants with normal alveolar ridge volume and resilience were included [3, 12, 24]. The residual ridge volume was considered normal when the contour of a cross-sectional portion of the edentulous ridges, on the individual CBCT scan, displayed a triangular shape, with the base ranging between the labial-buccal vestibules and the sides that correspond to the bilateral linear projection of both ridge slopes. Patients with highly resorbed ridges and knife-edge mandibular ridges were therefore excluded. Residual ridge resilience, evaluated using periodontal probe, was considered normal when it exhibited a displacement of approximately 2 mm. Individuals with residual ridges showing a displacement of more than 2 mm were, therefore, excluded [3, 12, 24]. The inclusion criteria are listed in Table 1. Eligible participants were allocated according to a sequence of computer generated random numbers (allocation ratio, 1:1:1). A researcher (M.A) who was not involved with other parts of the trial prepared the sequence code and transferred it to sealed envelopes. Details of the study were explained to all patients including the aims of the study, the number of visits, the level of cooperation needed, details about the materials used and all possible harms. Consequently, all participants read and signed the consent forms.

**Table 1:** Inclusion Criteria

1	Completely edentulous for at least 6 months with no previous denture wearing experience (first time complete denture wearers).
2	Aged 40–90 years
3	Normal maxillary and mandibular ridges [3, 12, 24]
4	No relevant medical conditions that can affect the course of the treatment including masticatory, neuromuscular, auditory or psychological conditions and no oral pathologies including lesions or ulcers, xerostomia, or tongue tie.
5	Read and signed the consent form

The fabrication of all dentures was performed by 2 experienced prosthodontists (N.E., Y.O.) and laboratory steps were performed by an experienced dental laboratory technician. After the primary impressions were made from impression compound modeling plastic material (Impression Compound; Kerr Corp., Orange, CA), they were sent to the dental laboratory where they were poured using Type III dental stone (Microstone; Whip Mix Corp., Louisville, KY) to produce preliminary casts, on which custom trays were fabricated from light-polymerizing resin sheets (Vertex Light Curing Trayplates; Vertex-Dental, Zeist, Netherlands). Custom trays were consequently border molded using impression compound modeling plastic (Kemco Tracing Sticks Green; Kemdent, Swindon, UK), and used for taking definitive impressions by using zinc oxide eugenol (Outline; Cavex, Haarlem, Netherlands). The impressions were then used to produce master casts on which record bases (Vertex Light Curing Trayplates, Vertex-Dental, Zeist, Netherlands) and wax occlusal rims (Cavex Set Up Regular Modeling Wax; Cavex, Haarlem, Netherlands) were fabricated which were later used for recording the maxillomandibular relationship. The casts were then mounted on an average value articulator (Gysi simplex OU-H3; COMATSU, Saitama, Japan), and tooth arrangement was done accordingly. After the clinical evaluation of the waxed dentures, they were sent to the dental laboratory where they were then flasked (Hanau Flask and Compress; Pearson Dental Supply Co, Sylmar, CA) and processed with heat-polymerizing acrylic resin (Lucitone 199; Dentsply Sirona, York, PA). After finishing and polishing of the dentures, they were then delivered and participants were given review appointments for post-delivery adjustments until the participants reported no further complaints. Participants were then given a review appointment after 1 month.

At the review appointments, after using the dentures for a month with no denture adhesives, the participants evaluated their satisfaction with the dentures on a horizontal 100- mm VAS based on the criteria suggested by Celebic and Knezovic-Zlataric [5, 25]. The form included 10 aspects including general satisfaction with the dentures, satisfaction with comfort, retention, ease of cleaning, speech, and mastication. Furthermore, participants filled out an Arabic translation of the oral health impact profile for edentulous patients (OHIP-EDENT) questionnaire which consists of 20 items, each scored on a 1 to 5 scale: 1 = “never,” 2 = “hardly ever,” 3 = “occasionally,” 4 = “fairly often,” and 5 = “very often.” The total OHIP-EDENT score was calculated as the sum of the scores for all items, ranging from 20 to 100, where 20 is the best possible score and 100 is the worst possible score [1, 5, 26, 27].

Participants were subsequently given 1 type of denture adhesives according to the allocated group found in the sealed envelope. The C group received Corega Ultra denture adhesive (GlaxoSmithKline Brazil Ltda., Rio de Janeiro, Brazil), the O group received OlivaFix denture adhesive Cream (Bonyf, Heiligkreuz 16, Vaduz, Liechtenstein) and S group received Sea.bond denture adhesive seals (Sea.Bond, White planes, NY, USA). Adhesives used and their composition are shown in Table 2.

**Table 2:** Denture adhesive materials used in this study, their manufacturers and composition

Material	Manufacturers	Composition
Corega Ultra fresh 3d hold denture adhesive	(GlaxoSmithKline Brazil Ltda., Rio de Janeiro, Brazil)	Calcium/sodium PVM/MA copolymer, petrolatum, cellulose gum, parafinum liquidum, aroma, CI 73360, CI 15850:1. Does not contain zinc
OlivaFix	Bonyf, Heiligkreuz 16, Vaduz, Liechtenstein	Cellulose gum, Olive fruit oil, Calcium/Sodium PVM/MA copolymer, hydrogenated soybean oil, Trihydroxystearin, Silica, Menthol, Lecithin, *Citrus Limon* peel oil, Menthyl Lactate
Original Sea-Bond® Seals	Sea.Bond, White planes, NY, USA	Fabric (Cellulose Acetate and Rayon), PEG-90 M, Cellulose Gum, Algin, Chlorophyllin-Copper Complex, Red 40 Lake

Participants were given instructions for using and cleaning the denture adhesives. Participants in group C were instructed to apply the adhesive on clean and dry dentures, once a day, away from the edges as shown in the diagram on the adhesive package, rinse mouth before use, press dentures in place firmly and bite down for few seconds to secure hold. For adhesive removal, participants were instructed to remove the adhesive using warm water and soft brush and use COREGA Cleanser (GlaxoSmithKline Brazil Ltda., Rio de Janeiro, Brazil) for more thorough cleaning.

Participants in group O were given similar instructions, according to the leaflet provided with the adhesive and were instructed to clean mouth and dentures properly if adhesive residues remain using a clean paper towel. Participants in group S were instructed to place dry Sea. Bond seals, white side up, onto clean dry dentures, trim any overlap with scissors, lightly moisten and gently tap seal with fingertips, put denture in mouth and bite down evenly for 5 seconds. Participants were instructed to remove the seal at the end of the day by lifting the corner and peeling it away and to change seals daily. During the month of using the adhesive, weekly phone calls were made to each participants to ensure their compliance with the instructions provided.. All participants were finally reviewed after 1 month to fill out the same forms again.

**Fig. 1:**
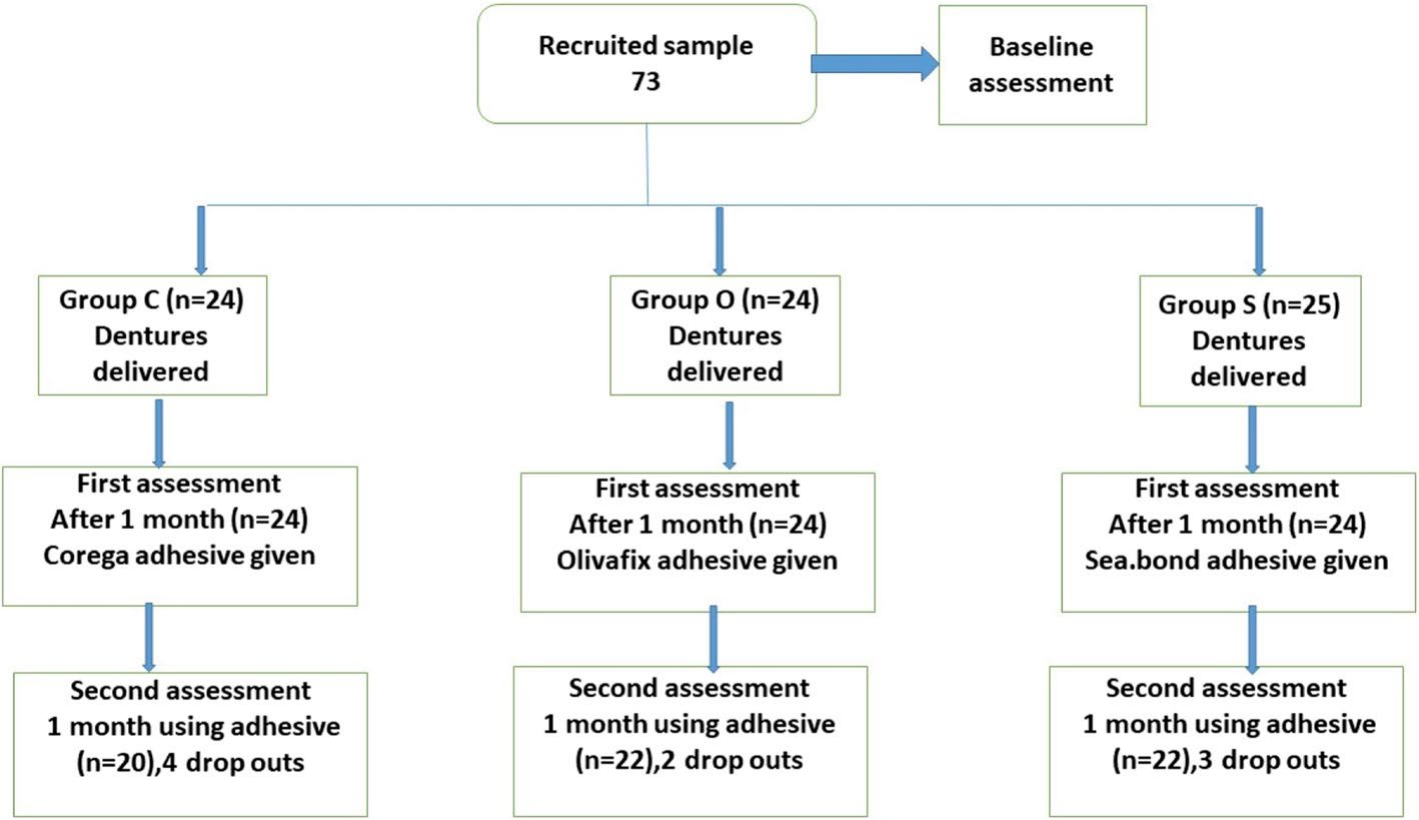
Study flowchart showing the numbers of participants in each group during the different stages of the study

Dentists who performed this study were blinded to which type of denture adhesive was given to the participants. Data collection was done by a dentist who did not participate in the treatment and was unaware of the allocated groups. A total of 60 participants were needed to detect a difference of 10 mm on the 100-mm VAS with 90% power (a = .05). To compensate for potential dropouts, 73 patients were originally included in the study, among which 9 were lost after they were given the denture adhesives. 64 participants were eventually included, with C group = 20, O group = 22, and S group = 22. This is illustrated in the flow chart in Fig. 1.

Statistical analyses were conducted by using a statistical software package (IBM SPSS Statistics, v22.0; IBM Corp). The data were inspected for normality by using the Kolmogorov-Smirnov and Shapiro-Wilk tests, and the results indicated that both VAS data and OHIP-EDENT were not normally distributed (*P* < 0.05). Therefore, Mann-Whitney U test was used to compare VAS readings before and after using the adhesive in each group separately and Wilcoxon signed-rank test was used to compare OHIP-EDENT scores within each group. The Kruskal Wallis test was then used to detect whether the effect of each adhesive in each group, which is the differences in the readings of VAS and OHIP before and after using the adhesive, was different among the 3 groups.

## Results

Seventy-three participants were initially recruited for this study. However, 9 of them didn’t show up for the second review appointment, resulting in a dropout rate of 12%. As a result, 64 participants were eventually included in this study. The sample consisted of 39 men (61%) and 25 women (39%). The age of the participants ranged between 48 and 83 years with Mean (M) ± standard deviation (SD) of 63.8 ± 9.3 years. Characteristics of the participants included in this study are illustrated in Table 3.

**Table 3:** Characteristics of the participants included in the study

Characteristics	Number of participants
**Gender**	
Male	39
Female	25
Age	
40–50	8
50–60	19
60–70	21
70–80	12
80–90	4
**Profession**	
Retired	12
Employed	20
Unemployed	32
**Education**	
Illiterate	2
Incomplete primary	4
Primary graduate	13
Incomplete high school	11
High school graduate	20
Incomplete University	4
University graduate	10
**Marital status**	
Married	39
Single	11
Divorced	6
Widowed	8

The results of the statistical analyses of this study are illustrated in Tables 4, 5, 6 and 7. Table 4 shows significant differences in all VAS values after using the adhesive within all groups, except for the ease of cleaning for Group O and Group S (*P* > .05). All 3 adhesives affected the ease of cleaning negatively, indicated in the table by negative values for the differences in VAS scores before and after using the adhesive, but this negative effect was only significant in C group. Table 5 shows that the total OHIP values after using the adhesives were significantly lower in all groups and some OHIP items were significantly lower after using the adhesives in all groups. The analyses of the differences in changes in VAS scores and OHIP-EDENT scores after using the adhesives between the 3 groups are illustrated in Table 6 and Table 7, respectively. Table 6 shows that the differences in VAS scores after using the adhesives were not significantly different between the 3 types of adhesives (*P* > .05), except for the ease of cleaning which was significantly different between Group C and Group S (*p* = 0.005). Regarding the OHIP scores, these differences between the 3 groups were all statistically insignificant (*P* > .05) as illustrated in Table 7.

**Table 4:** Mann – Whitney U test results showing medians and standard deviations for within group analyses of the differences in VAS scores before and after using the adhesives. * Indicates statistical significance at *p* = 0.05

Item	Group C	Group O	Group S
General satisfaction	9.0 ± 5.6*	9.0 ± 4.9*	10.0 ± 4.7*
Satisfaction with maxillary dentures	9.0 ± 3.4*	8.0 ± 2.4*	11.0 ± 3.5*
Satisfaction with mandibular dentures	18.0 ± 6.6*	12.0 ± 5.7*	10 ± 3.9*
Satisfaction with comfort maxillary	21.0 ± 7.1*	22.0 ± 6.9*	22.0 ± 8.2*
Comfort mandibular	19.0 ± 6.8*	15 ± 4.3*	11.0 ± 5.3*
Retention maxillary	9.0 ± 3.3*	11.0 ± 3.7*	10.0 ± 4.2*
Retention mandibular	22.0 ± 6.1*	21 ± 7.2*	16 ± 5.4*
Ease of cleaning	−20 ± 6.4*	−9.0 ± 3.4	−3.0 ± 1.5
Chewing	16.0 ± 5.0*	12 ± 5.6*	13 ± 4.1*
Speech	8.0 ± 2.7*	9.0 ± 3.1*	10.0 ± 2.4*

**Table 5:** Wilcoxin-Signed rank test results showing medians and standard deviations for within group analyses of the differences in OHIP scores before and after using the adhesives. * Indicates statistical significance at *p* = 0.05

Item	Group C	Group O	Group S
Difficulty in chewing any food	−1.0 ± 0.43 *	−1.0 ± 0.67 *	−1.0 ± 0.46*
Food catching in your dentures	−1.0 ± 0.62*	− 0.5 ± 0.25 *	−1.0 ± 0.22*
Dentures not fitting properly	− 0.5 ± 0.21 *	−1.0 ± 0.53*	−1.0 ± 0.57*
Painful aching in your mouth	− 1.0 ± 0.44*	− 0.5 ± 0.34*	−1.0 ± 0.45*
Uncomfortable to eat any food	− 1.0 ± 0.76*	−1.0 ± 0.68*	−1.0 ± 0.43*
Sore spots in your mouth	− 1.0 ± 0.55*	−1.0 ± 0.22*	−1.0 ± 0.23*
Uncomfortable dentures	−0.5 ± 0.41*	−0.5 ± 0.30*	−0.5 ± 0.33
Worried by dental problems	−0.5 ± 0.23	−0.5 ± 0.31*	−1.0 ± 0.44*
Self-conscious because of dentures	−0.5 ± 0.27*	0.0 ± 0.43	−0.5 ± 0.34*
Unclear speech	0.0 ± 0.21	−1.0 ± 0.48*	−1.0 ± 0.57*
Avoid eating some foods	− 1.0 ± 0.65*	−1.0 ± 0.54*	− 0.5 ± 0.47*
Unable to eat	−1.0 ± 0.55*	−1.0 ± 0.45*	−1.0 ± 0.74*
Interrupt meals	− 1.0 ± 0.62*	−1.0 ± 0.71*	−1.0 ± 0.53*
Upset with dentures	−0.5 ± 0.29*	−0.5 ± 0.31*	0.0 ± 0.51
Bit embarrassed	0.0 ± 0.23	0.0 ± 0.39	0.0 ± 0.40
Avoid going out	0.0 ± 0.36	−0.5 ± 0.37*	0.0 ± 0.50
Less tolerant of partner and family	0.0 ± 0.28	−0.5 ± 0.14*	0.0 ± 0.24
Irritable with other people	0.0 ± 0.62	−0.5 ± 0.19*	0.0 ± 0.61
Avoid other people company	0.0 ± 0.26	0.0 ± 0.43	0.0 ± 0.29
Feel life in general was less satisfying	−0.5 ± 0.31*	0.0 ± 0.28	0.0 ± 0.32
Total OHIP-EDENT	−10.5 ± 3.3*	−9.5 ± 2.5*	−10.5 ± 3.2*

**Table 6:** Results of Kruskal Wallis test showing *p* values for the analyses of between group differences in changes in VAS scores before and after using the adhesives

Item	Differences among groups *P* value
General satisfaction	0.211
Satisfaction with maxillary dentures	0.140
Satisfaction with mandibular dentures	0.106
Satisfaction with comfort maxillary	0.998
Comfort mandibular	0.331
Retention maxillary	0.476
Retention mandibular	0.063
Ease of cleaning	0.005 (Between C and S)
Chewing	0.209
Speech	0.276

**Table 7:** Results of Kruskal Wallis test showing *p* values for the analyses of between group differences in changes in OHIP-EDENT scores before and after using the adhesives

Item	Differences among groups (*P*-value)
Difficulty in chewing any food	0.09
Food catching in your dentures	0.23
Dentures not fitting properly	0.45
Painful aching in your mouth	0.26
Uncomfortable to eat any food	0.91
Sore spots in your mouth	0.61
Uncomfortable dentures	0.95
Worried by dental problems	0.61
Self-conscious because of dentures	0.56
Unclear speech	0.07
Avoid eating some foods	0.63
Unable to eat	0.39
Interrupt meals	0.11
Upset with dentures	0.18
Bit embarrassed	0.58
Avoid going out	0.68
Less tolerant of partner and family	0.19
Irritable with other people	0.20
Avoid other people company	0.89
Feel life in general was less satisfying	0.43
Total OHIP-EDENT	0.97

## Discussion

This study aimed to investigate the effects of different types of denture adhesives on complete denture patient satisfaction and oral health-related quality of life. The results showed that the use of denture adhesives significantly improved patient satisfaction scores and OHIP scores in all 3 groups, except for the ease of cleaning of the dentures before and after adhesive use in groups O and S. However, there were no significant differences in these effects between the 3 groups, except for the difference in ease of cleaning between groups C and S.

The findings of the present study were consistent with those of previous studies that reported positive effect for using denture adhesives [4, 6, 19]. However, Ohwada et al. 2019 findings presented conflicting results, suggesting no significant difference in OHIP-EDENT scores when adhesives were employed. The difference in their results could potentially be attributed to the duration of their study, which spanned only four days, as opposed to the present study where adhesives were used consistently for a month.

In this study, allocation of the participants was conducted by a coordinator so that the evaluators could be blinded [2]. Dentures were constructed by 2 dentists of similar experience who were blinded to patient allocation. However, the participants were not blinded because they were aware of the formulation for their allocated group. Nonetheless, all participants were first time denture wearers and thus had little or no experience with denture adhesives. Therefore, the chances for bias in different adhesive groups were low [28].

In our study, we did not find any significant differences in perceived adhesive effectiveness between the types of adhesives that were tested. This outcome stands in contrast to prior research that highlighted distinctions between various adhesive types [16, 18, 28]. The lack of differences between the different products investigated in this study might potentially facilitate a broader selection for consumers, allowing them to choose a denture adhesive based on personal preference and product availability. This broader range of options could potentially enhance acceptance and utilization among individuals who wear complete dentures [29].

The complete removal of adhesive from dentures is important for the hygiene and the stability of the dentures [30, 31]. Adhesives can be eliminated through mechanical means like brushing or through chemical methods employing water, soap solutions, or specific denture cleansers. It’s generally recommended to combine brushing with cleansers to ensure the thorough removal of adhesives from denture surfaces [30].

In our study, patients noted that Sea.Bond adhesive was notably easier to clean off the denture surface compared to Corega adhesive, attributed to its strip-based nature. This can make such type of adhesives an attractive option, especially for older people with impaired manual dexterity, especially that cream adhesives are usually transparent or pink in color and difficult to identify on denture surfaces [9, 30]. Our findings indicated no significant difference in the ease of cleaning between Corega and Olivafix, which is consistent with results previously observed by Polychronakis [30]. However, Olivafix was found to be easily solubilized by denture cleansers due to its oil-based components which were also found to have antioxidant, anti-inflammatory, and antimicrobial properties and hence Olivafix can inhibit growth of microorganisms even if adhesive remnants were not completely removed, making Olivafix another attractive option for denture wearers [32].

As the correlation between objective assessments and patient satisfaction has been reported to be poor [27], patient satisfaction is currently the decisive factor regarding the overall success of prosthodontic treatment and is therefore an important factor to justify the use of adhesives [3, 4]. Many studies investigated the experience of adhesive usage through questionnaires or VAS and nearly all of them reported positive outcomes [18]. Satisfaction outcomes are easy to measure, allow direct quantification of patients’ opinions and feelings towards different aspects of prosthodontic treatment and were found to be positively associated with oral health-related quality of life [8, 33]. The Oral Health Impact Profile for Edentulous Patients (OHIP-EDENT) has demonstrated a high correlation with each aspect of denture satisfaction. However, it is important to distinguish between oral health-related quality of life and satisfaction as two distinct outcomes [33]. Therefore, in our study we used both VAS and OHIP-EDENT as they complement each other in the assessment of the treatment outcome [1, 8, 19].

One of the limitations of this study was that only one follow-up measurement was considered although masticatory performance, patient satisfaction, and oral health-related quality of life are expected to improve with time [3]. However, studies showed that adhesives can improve subjects’ ability to adapt to conventional dentures during the adaptation period after denture insertion and this effect was limited after three months and participants often discontinue using the adhesive as it was found less convenient to use and hard to clean [9]. Therefore, studies investigating the long-term use of denture adhesives could be of limited value as long-term use of adhesives might be beneficial only in limited circumstances especially when clinical conditions are not favorable to denture rehabilitation, such as damaged tissues, xerostomia, or when the patients present decreased learning capability or poor neuromuscular coordination [9].

The extent of bone resorption has been highlighted in previous studies as a factor influencing satisfaction with dentures and OHIP-EDENT [3, 19]. Adhesive strength of denture adhesives to the basal seat mucosa and denture base resin might not be sufficient to improve the retention for severe residual ridge resorption [34]. Da Silva 2019 et al. found no influence of adhesive use on masticatory performance of participants with resorbed ridges and a negative influence on oral health related quality of life [22]. Hence, denture adhesives do not invariably improve denture function, irrespective of denture quality or condition of the denture-bearing tissue [5]. Therefore, severely resorbed mandibular ridges were excluded from the present study. However, future studies should include participants with more complex conditions such as resorbed or flabby ridges.

The duration of denture use can influence the subjective assessment of new dentures. Prior studies have shown that patients with previous denture experience tend to exhibit greater satisfaction with new dentures, while individuals who have been edentulous for a short period or are first-time denture wearers may express higher dissatisfaction with new dentures [3, 8].Therefore, only first time denture wearers were included in this study to minimize confounding factors. Furthermore, a period of adaptation is important before starting any treatment, therefore patients were given dentures with no adhesive for one month in our study for adaptation [3].

Our results showed that general satisfaction as well as all other aspects were improved by the use of denture adhesives. Therefore, it can be concluded that the improvement of general satisfaction here was a result of improvement of all factors including satisfaction with mandibular and maxillary dentures and factors such as esthetics, speech, stability, comfort, and the ability to chew. This disagrees with previous studies which concluded that only mandibular dentures influenced directly the overall satisfaction of denture wearers as their retention and comfort were improved after adhesive use and that speech and esthetics were not affected by adhesive use [3, 18, 22]. Speech is a complex skill that requires a prolonged period of adaptation, and a lack of improvement may be due to the short review period. The improvement in our study in all aspects suggest that the period of evaluation was sufficient for the patients to notice differences caused by the use of adhesives [3].The results also show that a significant improvement was found in the masticatory ability of patients, suggesting that the use of adhesives can improve functional features and did not affect the occlusal relationship of existing dentures [3].

Denture adhesives were reported in some previous studies to have some negative aspects including increased residual ridge resorption, development of oral diseases such as denture stomatitis and candidiasis especially in patients with xerostomia [14, 28]. However, short term use of denture adhesives was not reported to negatively alter oral microorganisms. Scientific evidence supporting the claim that properly used denture adhesives can cause oral pathologies, excessive bone resorption, or adverse changes in a patient’s occlusion is lacking [11, 15]. Since evidence regarding the effects of denture adhesives on the oral tissues when used for periods longer than six months is lacking, extended use of denture adhesives should not be considered without professional periodic assessments [11, 35]. As benefits of the use of denture adhesives tend to outweigh the negative effects, adhesives can be recommended for denture wearers according to the directions of the manufacturers and as long as good oral and denture hygiene are maintained [18]. Patients need to be informed that the use of denture adhesives cannot replace the need for a well-fitted complete denture and they need to be monitored periodically to evaluate prosthetic maintenance requirements [10]. Patients should be instructed to use the minimum amount necessary and to completely remove the adhesive from the prosthesis and the oral cavity on a daily basis [11, 35]. The majority of prosthodontic educators acknowledge the beneficial role of denture adhesives when used properly and current American Dental Association guidelines on the use of the denture adhesives should be integrated into both denture wearer education and the predoctoral dental curriculum [15]. With additional clinical trials and larger sample sizes, the credibility of using denture adhesives can be strengthened and standards stipulating the indications, proper instructions can be established. In this case, the use of denture adhesives can be regarded as a reliable treatment adjunct that can be incorporated to maximize the quantity and quality of health benefits, especially for patients with constrained budgets [1].

## Conclusions

Within the limitations of this study, the following conclusions can be withdrawn:

1. Using denture adhesives for completely edentulous patients resulted in higher patient satisfaction as indicated by higher VAS scores as well as improved quality of life as indicated by lower OHIP-EDENT scores after using the adhesives.
2. These improvements were not dependent on the type of adhesive, except for ease of cleaning as adhesive strips were easier to clean than paste type adhesives.

## Data Availability

This article has all the data that were collected or analyzed during this study.

## Abbreviations

VAS: Visual Analog Scale
OHIP EDENT: Oral health impact profile for edentulous patients
C: Corega
O: Olivafix
S: Sea.Bond
CONSORT: Consolidated Standards of Reporting Trials
M: Mean
SD: Standard Deviation
